# Outcomes of a Pilot Newborn Screening Program for Spinal Muscular Atrophy in the Valencian Community

**DOI:** 10.3390/ijns11010007

**Published:** 2025-01-14

**Authors:** Alba Berzal-Serrano, Belén García-Bohórquez, Elena Aller, Teresa Jaijo, Inmaculada Pitarch-Castellano, Dolores Rausell, Gema García-García, José M. Millán

**Affiliations:** 1 Cellular, Molecular and Genomics Biomedicine Group, La Fe Health Research Institute, 46026 Valencia, Spain; alba_berzal@iislafe.es (A.B.-S.); belen_garcia@iislafe.es (B.G.-B.); aller_ele@gva.es (E.A.); jaijo_ter@gva.es (T.J.); 2 Joint Unit CIPF-IIS La Fe Molecular, Cellular and Genomic Biomedicine, 46026 Valencia, Spain; 3 Center for Biomedical Network Research on Rare Diseases (CIBERER), Carlos III Health Institute, 28029 Madrid, Spain; 4 Department of Genetics, La Fe University and Polytechnic Hospital, 46026 Valencia, Spain; 5 Neuropediatric Unit, Department of Paediatrics, La Fe University and Polytechnic Hospital, 46026 Valencia, Spain; pitarch_inmcas@gva.es; 6 Clinical Analysis Service, Metabolic Disorders Laboratory, La Fe University and Polytechnic Hospital, 46026 Valencia, Spain; rausell_dol@gva.es

**Keywords:** spinal muscular atrophy, *SMN1*, newborn screening, dried blood spot, multiplex qPCR

## Abstract

Spinal muscular atrophy (SMA) is a degenerative neuromuscular condition resulting from a homozygous deletion of the survival motor neuron 1 (*SMN1*) gene in 95% of patients. A timely diagnosis via newborn screening (NBS) and initiating treatment before the onset of symptoms are critical for improving health outcomes in affected individuals. We carried out a screening test by quantitative PCR (qPCR) to amplify the exon seven of *SMN1* using dried blood spot (DBS) samples. From October 2021 to August 2024, a total of 31,560 samples were tested in the Valencian Community (Spain) and 4 of them were positive for SMA, indicating an incidence of 1/7890. Genetic confirmation was performed using multiplex ligation-dependent probe amplification (MLPA) and AmplideX PCR/CE *SMN1/2* Plus kit, in parallel obtaining concordant results in survival motor neuron 2 (*SMN2*) gene copy number. Within the first few weeks of their lives, two of the four patients detected by NBS showed signs of severe hypotonia, becoming ineligible for treatment. The other two patients were the first presymptomatic patients with two copies of *SMN2* to receive treatment with Risdiplam in Spain. In order to treat positive cases in their early stages, we conclude that the official deployment of SMA newborn screening is necessary.

## 1. Introduction

Spinal muscular atrophy (SMA) is the most prevalent genetic condition contributing to infant mortality, with an incidence around 1/6000–/10,000 live births and a carrier frequency ranging from 1/40 to 1/60 [1,2]. It is characterized by proximal muscle weakness and atrophy, areflexia, respiratory complications, among others. Historically, SMA has been classified into four types based on the age of onset and symptom severity. SMA type I (OMIM #253300), which accounts for around 60% of cases, typically presents before six months of age and follows an aggressive clinical progression. These patients do not achieve head support and may require permanent ventilation or face mortality by the age of two years if untreated [3]. SMA type II (OMIM #253550) usually develops between 6 and 18 months of age. Patients are able to sit and, in the absence of treatment, die between two years of age and young adulthood. Patients with SMA type III (OMIM #253400) manifest clinical signs after 18 months, typically in childhood or adolescence, and are able to stand and walk, although they lose these abilities in adolescence. Finally, an adult form of SMA type IV (OMIM #271150) has been described. The age of onset is highly variable, between 20 and 60 years old, and the disease has a slow progression characterized by progressive muscle weakness [4].

Recent advancements have refined this classification to include ten distinct clinical subtypes [5]. Additionally, a simpler categorization divides patients into three groups based on motor milestones: non-sitters, sitters, and walkers [6].

At the genetic level, SMA is an autosomal recessive disease caused by alterations in the survival motor neuron 1 gene or *SMN1* (OMIM #600354; NM_000344), in the chromosomal region 5q13.2 [7]. Biallelic *SMN1* deletion or gene conversion is the underlying molecular etiology of SMA in about 95% of affected people. Other type of mutations that cause *SMN1* loss of function can be seen in the remaining 5% of SMA patients [8,9,10]. Another gene linked to this condition is survival motor neuron 2, or *SMN2* (OMIM #601627; NM_017411). This gene is a paralog of *SMN1*, differing by just 20 nucleotides but codifying for the same protein. The unique difference in the coding sequence between both genes is the transition c.840C>T in exon seven of the *SMN2* gene that causes exon skipping in most *SMN2* pre-mRNA transcripts. This leads to the production of a truncated, nonfunctional protein (SMN-Δ7), which is quickly degraded post-translationally [11]. Due to the susceptibility of this region of chromosome five to genetic rearrangements, the number of *SMN2* copies varies widely among individuals, ranging from as few as zero to as many as eight. The degree of severity in SMA is mainly determined by the number of *SMN2* gene copies. Individuals with two copies typically experience the most severe form, while those with four or more copies tend to have a milder presentation [12,13]. Nevertheless, there appears to be a lack of perfect association between the *SMN2* copy number and clinical symptoms, suggesting the presence of additional phenotype-modifying variables [14,15,16,17].

The advent of disease-modifying therapies that change the natural progression of SMA has greatly enhanced patient outcomes. Two current treatments, Nusinersen and Risdiplam, are focused on enhancing the inclusion of exon seven in *SMN2* to produce a functional, full-length SMN protein from *SMN2* mRNA. The third treatment, Onasemnogene Abeparvovec, is a gene replacement therapy that delivers a copy of the functional human *SMN1* cDNA using the adeno-associated virus nine (AAV9) as a vector [18,19,20].

Early intervention is crucial due to the rapid progression of SMA. The most favorable treatment outcomes are achieved when therapy is initiated before or shortly after symptom onset. This urgency has accelerated the global adoption of SMA newborn screening (NBS) programs [20,21,22,23,24,25,26,27,28,29,30,31,32,33].

The aim of this study is to provide a presymptomatic diagnosis of SMA, allowing for early therapeutic intervention, and to promote the inclusion of SMA in the official newborn screening program of the Valencian Community.

## 2. Materials and Methods

### 2.1. Valencian Pilot Project

The pilot project was conducted in the La Fe Health Research Institute in Valencia between October of 2021 and August of 2024 and was divided in two phases: phase I from October of 2021 to March 2023 and phase II from March 2023 to August 2024. In phase I, only two hospitals participated in the study: at first, exclusively La Fe University and Polytechnic Hospital of Valencia (phase Ia), and from May of 2022, also the University Clinical Hospital of Valencia (phase Ib). In March 2023, the second phase began thanks to a new plan of the Public Health Department of the Valencian Community that allowed the storage of the residual newborn screening blood spot samples in the Biobank for the Biomedical and Public Health Research of the Valencian Region for research use. With parental informed consent, this proposal allowed the transfer of excess screening samples for scientific research. From then on, all newborns in the Valencian Community whose parents signed the informed consent had the possibility to be screened for SMA.

The study was conducted according to the guidelines of the Declaration of Helsinki and approved by the ethics committee of La Fe Health Research Institute (project number 2020-517-1 “Newborn screening for spinal muscular atrophy”/21-10-2020). All parents of the subjects provided their informed consent before being included in the study. In each phase of this study, a different and specific informed consent was required to screen any newborn for SMA.

### 2.2. Patients and Samples

The maternity department notified parents of the potential of participating in the SMA newborn screening and offered informed consent for this study at the same time as they signed the general newborn screening informed consent.

### 2.3. Newborn Screening Methodology

Dried blood spot (DBS) circles with a diameter of 3.2 mm were cut and placed in a 96-well plate using the collection device Panthera DBS Puncher instrument (Revvity). DNA was isolated through the JANUS G3 workstation instrument (Revvity) in a semi-automated manner. The extraction protocol was performed using the Eonis DNA Extraction kit (Revvity) and comprised two washing steps of the DBS samples at 25 °C and 700 rpm, followed by an elution step at 70 °C and 700 rpm.

Reagents from the Eonis SCID-SMA kit (Revvity) were used for quantitative PCR (qPCR), which allowed the detection of SMA and severe combined immunodeficiency (SCID) simultaneously, although the project focused exclusively on the SMA disease. This kit contained a specific probe for exon seven of *SMN1* and another for *RPP30*. The *RPP30* gene was employed as an internal amplification control for DNA quality and quantity. The assay was monitored by analyzing one analyte-negative control and two *SMN1*-positive controls, all three with the same *RPP30* levels, and no template control. To start the qPCR process, 3 uL of eluted DNA were added to a new 96-well plate containing 12 uL of the qPCR reagents (6 uL of each reagent). The qPCR protocol was carried out in the QuantStudio Dx Real-Time PCR instrument (Thermo Fisher, Waltham, MA, USA) and the program consisted of 37 °C for 2 min, 94 °C for 5 min and 40 cycles of 93 °C for 10 s, 60 °C for 30 s, and 69 °C for 40 s.

According to the qPCR kit specifications, presumptive normal samples were defined as those having the cycle threshold (Ct) value between 15 and 32 for *RPP30* and between 15 and 31.24 for *SMN1*. Thus, a Ct value for *SMN1* above 31.24 would indicate presumptive positive for SMA. In the presumptive positive cases, the same qPCR protocol was repeated in the original DBS sample in duplicate. In cases where the absence of *SMN1* exon seven was found once again, referral was made to the Neuropediatric Unit of La Fe University and Polytechnic Hospital for anamnesis and clinical evaluation and to the Genetics Department of La Fe University and Polytechnic Hospital for the genetic confirmation. With this technology, only cases with zero copies of *SMN1* were detected and reported.

### 2.4. Clinical Assessment

**Figure 1:**
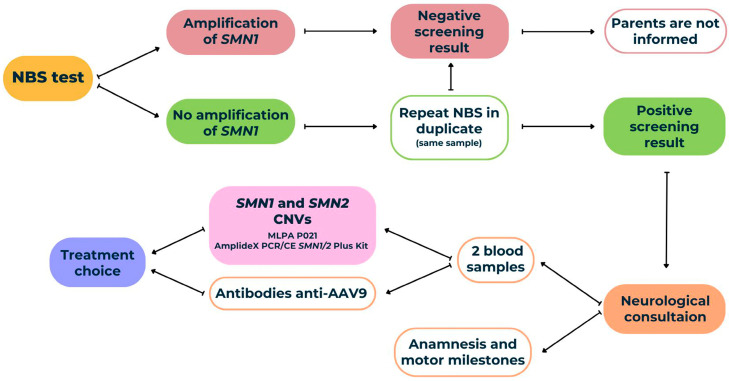
The Valencian community’s spinal muscular atrophy (SMA) pilot project screening pipeline. After obtaining a positive screening result twice, the neuropediatric unit schedules a visit with the proband’s family to conduct the motor tests and to take two blood samples. One of them is to detect the presence of anti-adeno-associated virus 9 (AAV9) antibodies and the other to quantify the copy number of the survival motor neuron 1 gene (*SMN1*) and survival motor neuron 2 gene (*SMN2*) by two techniques. Based on these two results, a treatment plan is chosen and started right away.

In screen positive cases, the affected families were initially contacted by the Neuropediatric Unit of La Fe University and Polytechnic Hospital for consultation. Upon arrival at the hospital, the families were informed of the results, about the disease, and possible treatment options. The clinical assessment focused mainly on motor function, but also on respiratory and swallowing functions. In terms of motor function, the CHOP INTEND (Children’s Hospital of Philadelphia Infant Test of Neuromuscular Disorders) scale was used, and the neurophysiological assessment was carried out through the study of motor conduction by CMAP (Compound Motor Action Potential). A basic blood test (hemogram, biochemistry, and coagulation) was also performed to assess troponin T as a marker of cardiac damage, among others. Lastly, two blood samples were obtained: one for testing anti-AAV9 antibodies and the other for DNA extraction and confirmation of the screening result (Figure 1). All these clinical practices are contained in a consensus guideline [34].

### 2.5. Diagnostic Confirmation

A fresh blood sample was obtained during the first consultation with the Neuropediatric Unit and tested in the Genetics Department of La Fe University and Polytechnic Hospital for diagnostic confirmation (Figure 1). To determine the copy number of *SMN1* and *SMN2* simultaneously, two confirmatory tests were used in parallel: Multiplex Ligation-dependent Probe Amplification (MLPA) with SALSA Probemix P021-B1 (MRC Holland) and the AmplideX PCR/CE *SMN1/2* Plus Kit (Asuragen) [35,36,37]. Gene rearrangements, gene conversions, and hybrid genes between *SMN1* and *SMN2* can be detected through MLPA. The AmplideX PCR/CE *SMN1/2* Plus kit allows the quantification of *SMN* gene copy number too, as well as three relevant variants for the pathology: the c.*3+80T>G and c.*211_*212del variants in *SMN1* associated with carriers 2/0 (two copies of *SMN1* in the same allele) and the c.859G>C variant in *SMN2* associated with a milder phenotype [16,38,39,40].

## 3. Results

### 3.1. Screening for SMA

In the Valencian Community, 103,594 infants were born between October 2021 and August 2024, of which 31,560 were screened for SMA (30.5%). In particular, 6411 patients were screened in the first phase and 25,149 in the second.

Given that, currently, there are about 35,000 births per year in the Valencian Community, the estimated incidence of SMA ranges from 1 in 10,000 to 1 in 6000 newborns, and according to historical records from the Genetics Department of La Fe University and Polytechnic Hospital from 2001 to the present, three or four new cases per year are expected [1,2]. Of the 31,560 samples tested, 4 of them had screen positive for SMA; so, the incidence observed in this study was 1/7890 (4/31,560). These results are in line with other pilot SMA screening programs [21,22,23,24,25,26,27,28,29,30,31,32,33] and are summarized in Table 1.

The four positive cases detected by neonatal screening were confirmed by MLPA, and the AmplideX PCR/CE *SMN1/2* Plus kit showed the same result; so, no false-positive screening result was found. To our knowledge, we did not identify any false-negative screening result to date. As a result, the method’s sensitivity and specificity were 100%. As 40 samples were repeated due to either issues with the Janus equipment (29) or the lack of *RPP30* amplification (11), the repeatability rate was 0.1267% (40/31,560).

**Figure 2:**
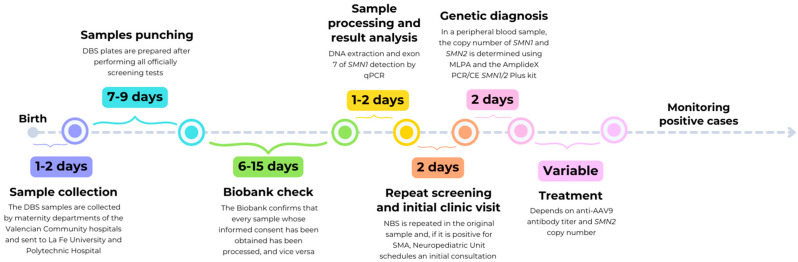
Timeline and outcome of positive cases in the Valencian SMA newborn screening (NBS) program. The entire time between sample collection and SMA screening was delayed from 15 to 30 days.

As this was a research project, the time elapsed from the newborn sample collection to the release of the results was much longer than that for official newborn screening. The first limitation was the late access to the samples, which were available between seven and nine days after collection. The second limitation was related with the Biobank acting as an intermediary between the laboratory that received the samples and the laboratory that performed all of the technical part. The Biobank reviewed each informed consent, which led to a delay of up to 15 days in the delivery of the samples. In summary, there was a total delay from 15 to 30 days from the time the sample was collected to when the SMA screening was performed. Figure 2 summarizes the time spent on sample collection, screening, diagnosis, therapy, and monitoring of SMA NBS.

### 3.2. Genetic Diagnosis and Clinical Management of Screen Positive Cases

The pilot project of SMA newborn screening led to the diagnosis of four positive cases between 15 and 30 days of life (cases III, V, VI, and VII in Table 2). All patients obtained the same results when MLPA and the AmplideX PCR/CE *SMN1/2* Plus kit were used, with case III with one copy of *SMN2* and cases V, VI, and VII with two copies of *SMN2*. None of the cases under investigation had the positive c.859G>C mutation in *SMN2*.

**Figure 3:**
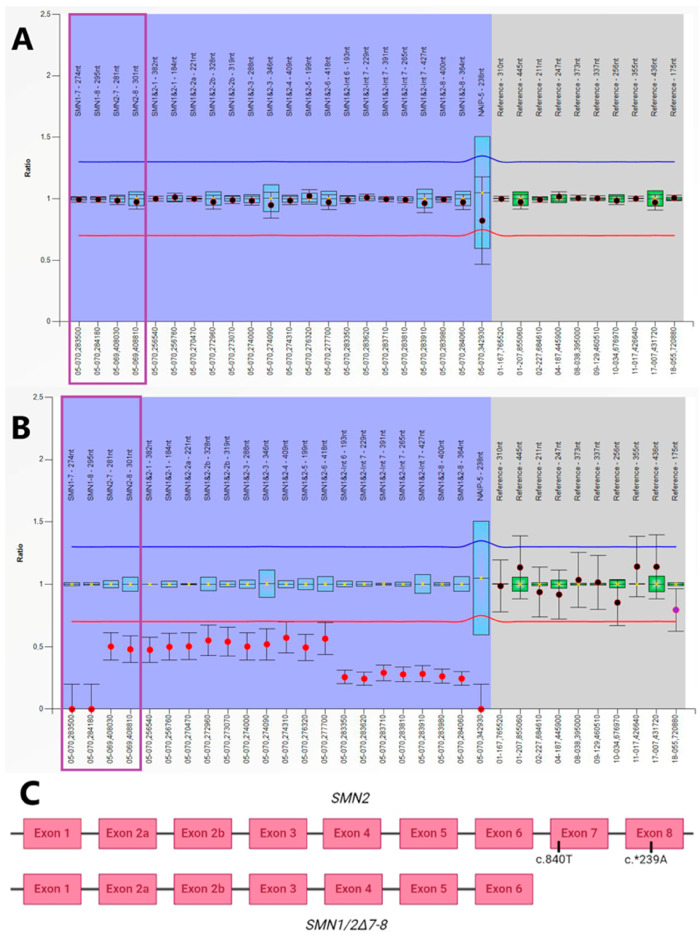
Multiplex ligation-dependent probe amplification (MLPA) results of *SMN1* and *SMN2* genes in a healthy control (**A**) and case III (**B**,**C**). The SALSA MLPA Probemix P021-B1 SMA contains 4 probes specifically designed to identify exons seven or eight of *SMN1* and *SMN2* (marked with a purple box) and 17 probes that detect sequences present in both *SMN1* and *SMN2*. In the instance of the four mentioned probes, rough ratio values of 0, 0.5, and 1 indicate homozygous absence (zero copies), heterozygous absence (one copy), and normal copy number (two copies), respectively. Patient III had zero copies of *SMN1* exons seven and eight and one copy of *SMN2* exons seven and eight (**B**). On the other hand, both *SMN1* and *SMN2* sequences hybridize with the remaining 17 probes. In control individuals carrying two copies of *SMN1* and two copies of *SMN2* (**A**), the 17 probes detect four gene copies in total (ratio 1). In patient III, these 17 probes detected two copies of the exons one to six of *SMN* genes, and one copy from intron six to exon eight of *SMN* genes (**B**). This means that the patient has one complete copy of *SMN2* and the other incomplete with only exons one to six marked as *SMN1/2*∆7-8. Additionally, patient III lacked exon five of the *NAIP* gene, which is also found at the *SMN* locus but unrelated to SMA. (**C**) represents the structure of *SMN* genes in patient III: a complete copy of *SMN2* and an incomplete copy *SMN1/2*∆7-8. Positions c.840 in exon seven and c.*2390 in exon eight are different between *SMN1* and *SMN2*.

The first case of SMA detected by newborn screening was case III (Table 2). Case III was a SMA type 0 with severe hypotonia, tongue fasciculations, arthrogryposis, and congenital cardiopathy from birth, so by the time of SMA neonatal screening (15 days), the patient had already died. MLPA revealed the presence of two copies of the *SMN* genes, one complete copy of *SMN2* and the other with a partial deletion from intron six to exon eight (Figure 3B). This incomplete copy had only exons one to six, which are identical between *SMN1* and *SMN2*, and was described as *SMN1/2*∆7-8 (Figure 3C) [35,41,42]. For practical purposes, this infant only had one copy of *SMN2*; therefore, none of the treatments would have been appropriate. In June 2024 and just two days apart, two cases of SMA (cases V and VI) were detected 20 days after birth. Both infants had two copies of *SMN2* and were asymptomatic at the time of diagnosis (Table 2). Risdiplam was the therapy of choice in both cases at one month of life due to the presence of anti-AAV9 antibodies in the blood in case V and to the parents’ decision in case VI. Both patients remain asymptomatic and attend regular neurological examinations.

Heading to the end of the pilot program, a fourth case was identified (case VII). Despite exhibiting severe hypotonia as early as the second week of life, SMA newborn screening was performed at one month of age (Table 2). Due to the patient’s early onset and the confirmation of two copies of *SMN2* through genetic testing, the patient was classified as type Ia. Palliative care was chosen by the parents due to the severity of the patient at the time of diagnosis.

Unfortunately, during the project period, three additional SMA patients (cases I, II, and IV) were born (Table 2). However, their samples could not be analyzed as a part of the project, and they were initially diagnosed based on the manifestation of clinical signs. Due to the lack of informed consent from their parents, these patients were not eligible for the SMA newborn screening project. Through the use of genetic testing methods, two copies of *SMN2* were discovered in these three patients. Cases I and IV had SMA type Ia with severe hypotonia before two weeks of age; so, no therapy could help them.

In contrast, despite the fact that case II could not be diagnosed presymptomatically, she was treated with gene therapy (Onasemnogene abeparvovec). This infant had newborn hypotonia at three months of age, which corresponds to SMA type Ib. Two copies of *SMN2* were found using the Amplidex PCR/CE *SMN1/2* Plus kit and MLPA analysis (Table 2). The patient is currently under observation and is showing positive progress.

The clinical and genetic information of the seven patients born during the study period are shown in Table 2, sorted by month of birth.

## 4. Discussion

Every year, approximately 35,000 new babies are born in the Valencian Community, where they are screened for 13 different disorders at the time of birth (congenital hypothyroidism, congenital adrenal hyperplasia, cystic fibrosis, phenylketonuria, maple syrup urine disease, classic homocystinuria, tyrosinaemia type 1, propionic/methylmalonic acidemia, glutaric acidemia type 1, medium chain acyl-CoA dehydrogenase deficiency, long-chain 3-OH acyl-CoA dehydrogenase deficiency, biotinidase deficiency, and sickle cell anemia). We conducted a pilot study for SMA screening from October 2021 to August 2024 in order to encourage its inclusion in the Valencian Community’s official newborn screening program, pending its inclusion in the National Health System’s common services portfolio.

A total of 31,560 infants were screened by quantitative PCR and homozygous absence of exon seven of *SMN1* was detected in 4 of them. Consequently, the observed incidence was 1/7890 (4/31,560), in line with previous SMA newborn screening programs like those in Germany (1/7524) or Latvia (1/9091) [24,29,43].

Three additional patients were born during the project period, but due to the lack of access to informed consent, they were not eligible for early SMA screening. No false-positive or false-negative screening results were detected, resulting in a clinical sensitivity and specificity of 100%, and the repetition rate was 0.1267% (40/31,560). However, if a patient with a mild clinical presentation was not detected, it will become evident in a few years because La Fe University and Polytechnic Hospital serves as the Valencian Community’s reference center for the administration of SMA treatment. In order to prevent the unintentional identification of carriers with a single copy of *SMN1*, the project was developed to detect only the homozygous *SMN1* absence. For this reason, the putative 5% of patients with other *SMN1* abnormalities were also undetected [8,9,10,44].

The timeframes for obtaining results significantly exceeded those required by official neonatal screening programs due to various factors mentioned previously, all of which are related to the fact that it was a research project rather than an official program (Figure 2). We obtained the analysis results within a range from 15 to 30 days after birth. In cases of SMA, even a delay of just a few days in diagnosis and treatment can significantly impact the prognosis and survival of patients, especially those with two copies of the *SMN2* gene.

Out of the 103,594 infants born between October 2021 and August 2024 in the Valencian Community, 31,560 were screened for SMA, representing 30.5%. Adherence to the project increased substantially thanks to the decision of the Department of Public Health to donate surplus screening samples to research projects, from 15.5% at the end of the first phase to 47.9% in the second phase. Nevertheless, complete project adherence was not attained. There were several explanations for the incomplete coverage. Firstly, not all hospitals offered informed consent for SMA screening. Secondly, although some centers offered informed consent, we understand that it may be challenging to adequately communicate the importance of participating in this research project, as well as for parents to understand the benefit or relevance of donating the surplus sample for the project. This is why there were instances where parents made an informed decision to decline participation in the study.

The exact *SMN2* copy number is crucial for therapy selection, as it influences treatment options. Given that discrepancies between methods have been reported [45,46], two technologies, MLPA and the Amplidex PCR/CE *SMN1/2* Plus kit, were used concurrently for post-screening genetic diagnosis. Employing both methods in parallel ensured greater accuracy in determining the *SMN2* copy number. In addition to the *SMN2* copy number, each method provides different and complementary information relevant to the pathology. MLPA enables the detection of deletions and duplications in *SMN1* and *SMN2*, as well as hybrid genes. The Amplidex PCR/CE *SMN1/2* Plus kit detects the c.859G>C variant in *SMN2*, which, while not critical for treatment decisions, plays a key role in the patient’s prognosis. The two approaches yielded identical numbers of *SMN2* copies in every patient, obtaining two copies in all patients, except for case III, with only one complete copy of *SMN2*. This was the only patient with an incomplete copy due to a rearrangement between the *SMN1* and *SMN2* genes (*SMN1-2*∆7-8).

Significant clinical differences were observed in the patients with the same *SMN2* copy number. Specifically, six of the seven patients studied had two copies of *SMN2* and phenotypes ranged from marked hypotonia at ten days of life and subsequent death to asymptomatic at two months of life. These variations in clinical manifestation were described previously [13,14,47] and could be a sign of additional phenotype modifiers, including positive or negative variants in *SMN2* or additional genes, like the plastin 3 gene (*PLS3*), or other not-yet-identified genes that could be modifying the phenotype [15,41,48,49,50]. In our case, none of the seven patients possessed the *SMN2* variant c.859G>C, which is linked to a milder phenotype.

Given their early presentation and pronounced hypotonia within days of birth, 50% of the individuals diagnosed through neonatal screening were categorized as SMA type 0/Ia, and the remaining 50% were identified during the presymptomatic phase. This high rate of severe clinical manifestations is consistent with a study that found 43.52% of patients in a Spanish cohort of 625 patients had a severe non-sitter phenotype [13].

Currently, there are three approved treatments for spinal muscular atrophy (SMA) in Spain: Nusinersen, Onasemnogene Abeparvovec, and Risdiplam. Nusinersen and Risdiplam are indicated for patients with two to four copies and one to four copies of the *SMN2* gene, respectively. Onasemnogene Abeparvovec is approved for patients with up to three copies of *SMN2*, aged less than nine months, with a body weight of up to 13.5 kg, and anti-AAV9 antibody levels below 1:50. In the presymptomatic phase, it is indicated for patients with two copies of *SMN2*, under six weeks of age, with a gestational age of 35–42 weeks, and anti-AAV9 antibody levels below 1:50. During the SMA newborn screening, two presymptomatic patients with two copies of the *SMN2* gene were found to be eligible for one of the treatments. In case V, elevated antibody levels excluded eligibility for Onasemnogene Abeparvovec, leading the parents to choose Risdiplam. In case VI, the parents ultimately choose to treat with Risdiplam, even though the patient was a candidate for all three medications. These two cases became the first two presymptomatic patients in Spain to receive Risdiplam.

## 5. Conclusions

This study demonstrates the feasibility and necessity of SMA newborn screening in the Valencian Community. Pilot research projects are needed to determine the true incidence of the disease, the sensitivity and specificity of the methodology, and the cost-effectiveness ratio. However, the research project requires additional informed consent signatures, which complicates families’ adherence to participate. In fact, in our project, we were only able to analyze, on average, 30.5% of the newborns during that period, emphasizing the importance of including SMA in official screening programs. The method of quantitative PCR provides a reliable, robust, sensitive, and specific approach to screen for SMA in neonates. Since there have been reports of variations in *SMN2* copy numbers across methods and their significance in treatment selection, using MLPA and the Amplidex PCR/CE *SMN1/2* Plus kit in parallel guarantees accurate diagnosis and the identification of the c.859G>C variant in *SMN2*, which significantly affects patient outcomes. The presymptomatic detection of SMA patients is key to more effective treatment, especially in the most severe clinical manifestations. Therefore, we hope that this pilot project will facilitate the inclusion of SMA in the official newborn screening program of the Valencian Community and, subsequently, in the common services portfolio of the Spanish National Health System and make possible faster patient care.

## Figures and Tables

**Table 1:** Summary of the global results of SMA newborn screening in the Valencian community. In phase I, a total of 6411 children born in two hospitals in Valencia were screened and no positive cases were found. In March 2023, phase II of the project started, extending SMA screening to all hospitals in the Valencian community. During this period, the study was only accessible to newborns whose parents had signed the informed consent. Four positive cases were detected (incidence 1/7890), but three other patients were unable to be screened because they did not have access to such informed consent.

Phase	Period	Hospitals Participating in the Project	Number of Births	Number of Newborns Analyzed	% of Newborns Screened	Number of Positive Cases Detected	Number of SMA Patients Born in This Period (to Our Knowledge)
Ia	October 2021–April 2022	La Fe University and Polytechnic Hospital	20,935	1728	8.25%	0	0
Ib	May 2022–February 2023	La Fe University and Polytechnic Hospital and University Clinical Hospital	30,152	4683	15.5%	0	0
II	March 2023–August 2024	All hospitals in the Valencian community	52,507	25,149	47.9%	4	7
	Total		103,594	31,560	30.5%	4	7

**Table 2:** Clinical and genetic data of patients born during the SMA newborn screening project period. (**A**) shows the patients’ month of birth, whether or not the parents signed an informed consent, and their genetic data. All patients obtained the same copy number of *SMN2* by MLPA and Amplidex PCR/CE *SMN1/2* Plus kit. One patient (case III) had an incomplete copy of *SMN1/2* (*SMN1/2*∆7-8), and no patient possessed the protective variant in *SMN2* c.859G>C (Amplidex PCR/CE *SMN1/2* Plus kit). (**B**) collects the clinical data. Clinical classification of the patients was conducted using the Cuscó et al. 2020 guideline [5]. Patients who were not candidates for any treatment (cases I, III, IV, and VII) were not tested for anti-AAV9 antibodies. The results of neonatal motor skills and motor conduction are also reported in the table. Troponin T values between 0.00 ng/L and 14.00 ng/L are considered non-pathological. NA: Not applicable; CHOP INTEND: Children’s Hospital of Philadelphia Infant Test of Neuromuscular Disorder; CMAP: Compound motor action potential.

A									
Case	Month of Birth		Informed Consent		Time Until NBS		*SMN2* Copy Number		
I	07/2023		No		-		2		
II	08/2023		No		-		2		
III	10/2023		Yes		15 days		1		
IV	01/2024		No		-		2		
V	06/2024		Yes		20 days		2		
VI	06/2024		Yes		20 days		2		
VII	07/2024		Yes		30 days		2		
**B**									
**Case**	**Onset of Clinical Signs**	**Type of SMA**	**Anti-AAV9 Antibodies**	**CHOP INTEND** **Motor Scale** **(0–64)**	**Ultrasensitive Troponin T** **(ng/L)**	**Right Ulnar CMAP** **(mV)**	**Treatment**	**Start of Treatment (Age)**	**Alive**
I	7 days	Ia	NA	0/64	141.00	0.0	Palliative care	-	No
II	90 days	Ib	No	21/64	19.40	0.4	Onasemnogene abeparvovec	135 days	Yes
III	0 days	0	NA	0/64	230.00	0.0	Palliative care	-	No
IV	15 days	Ia	NA	0/64	Undetermined	0.0	Palliative care	-	No
V	Asymptomatic	Presymptomatic	Yes (1:200)	38/64	Undetermined	0.6	Risdiplam	30 days	Yes
VI	Asymptomatic	Presymptomatic	No	40/64	69.80	2.4	Risdiplam	30 days	Yes
VII	10 days	Ia	NA	0/64	53.00	0.0	Palliative care	-	No

## Data Availability

Data are contained within the article.
